# Why the racing industry and equestrian disciplines need to implement population pharmacokinetics: To learn, explain, summarize, harmonize, and individualize

**DOI:** 10.1002/dta.3706

**Published:** 2024-04-29

**Authors:** Pierre‐Louis Toutain

**Affiliations:** ^1^ INTHERES Université de Toulouse, INRAE, ENVT Toulouse France; ^2^ The Royal Veterinary College University of London London UK

**Keywords:** horses, medication control, nonlinear mixed effect model, population pharmacokinetics, withdrawal time

## Abstract

Population pharmacokinetics (POP PK) is a powerful pharmacokinetic tool, which measures quantitatively, and explains the variability in drug exposure and drug effect between individuals. POP PK uses an observational (nonexperimental) approach; it is conducted in the target population living in its normal environment (e.g., farm and race‐track). The strength of the POP PK approach lies in its greater relevance for the population studied in its different natural environments than experimental studies carried out in more or less biased laboratory conditions. In clinical settings, it is commonly necessary to restrict the number of samples per subject collected for analysis and the derived data cannot be analyzed using traditional individual data analytical methods; rather data are merged and analyzed with an appropriate statistical tool: the nonlinear mixed effect model (NLMEM). POP PK modeling is frequently used with the objective of adjusting drug dosage, and hence drug exposure, not only for the whole population but also for subgroups of animals (e.g., for a given breed, sex, and age). It can also have application at the individual subject level, in the context of precision medicine. For horses, the use of the POP PK/PD model will allow prescribers to estimate an individual Withdrawal Time for a given horse whose treatment they are supervising. Another potential field of application will be meta‐analysis of existing data to generate new knowledge on a drug or to collate and synthesize, in an objective and transparent manner, existing data; this will facilitate harmonization of screening limits at an international level.

## Graphical abstract

Population pharmacokinetics (POP PK) is a pharmacokinetic tool, which measures and explains the variability in drug exposure and drug effect between individuals. For prescribers, POP PK model will allow to estimate an individual Withdrawal Time for a given horse. For scientists, POP PK model will allow meta‐analysis of existing data and to generate new drug knowledge. For organisations, POP PK model will allow to objectively and impartially summarize the data available to experts, facilitating consensus when establishing international screening limits.

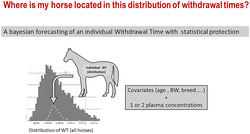

## 1 OVERVIEW OF POPULATION PHARMACOKINETICS (PK)

Data for both PK and pharmacodynamics (PD) are subject to variability; POP PK (or POP PK/PD) is a powerful observational approach, which aims to measure and explain the variability in drug exposure between individuals—humans or animals.^1^ In essence, every horse is unique. Horses differ from each other by their demographic characteristics, such as breed, sex, and age; by their managerial conditions of care (environment, habitat, diet); and by the sporting objectives assigned to them (racing, equestrian discipline, training level, and more). Thus, for all drugs, each horse is unique in terms of PK and PD profiles, and this interindividual variability must be identified, quantified, and possibly explained by the aforementioned characteristics. In this review, these are referred to as *covariates*.

The only means of studying, comprehensively and with minimal bias, the main factors determining PK and PK/PD variability is to investigate the drug directly in the target equine population. This necessitates conducting trials under field conditions. Therefore, POP PK is essentially an observational and nonexperimental clinical science. Given the observational nature of POP PK studies, it will often be impractical to collect multiple blood or urine samples from single horses. Typically, the collected data will be limited from one to four plasma/urine samples per horse. Moreover, they will generally be obtained at varying time schedules between horses to provide operational flexibility for clinicians conducting the trial. This kind of sampling is referred to as “sparse and unbalanced.” Data sets are considered unbalanced, when all study subjects do not contribute the same number of observations. In contrast, data obtained in an experimental setting are usually “rich and balanced,” because each horse can be sampled many times, without operational difficulties. Because of these fundamental differences in sampling schedules between experimental and observational contexts, data analyses necessarily differ also. Rich and balanced data can be, in a first step, analyzed individually (horse‐by‐horse). In a second step, possible correlations between individual PK parameter values and monitored demographic factors can be undertaken. This is referred to as *Two‐Stage Data Analysis* (TSA), and the vast majority of published PK and PK/PD data in horses has been obtained using this approach. When data are sparse and unbalanced, this approach is inapplicable, as each horse does not provide the information necessary to select a PK model for estimation of its parameters. Hence, observational data must be pooled for analysis by an appropriate statistical model known as the n*onlinear mixed effect model* (NLMEM) or NONMEM. This model is described in Section 3 of this review.

The population approach was introduced into human medicine in the 1970s for individualization of drug therapy.^2^ It is now routinely used in drug development programs, and procedural guidelines were issued by the US FDA^3^ and the EU EMA.^4^ Historically, the progression of POP PK in veterinary medicine has been very slow,^5^ although it has accelerated rapidly in recent years.^6^ There are two reasons for this: the sophisticated nature of data analysis and the difficulty for stakeholders both to understand its jargon and recognize the uniqueness of its contribution in addressing questions that cannot be solved with other approaches.

This review does not consider technical aspects of population analysis.^7^ The objective is rather to highlight the value, for the racing horse industry and equestrian disciplines, that can be derived from NLMEM in answering a range of questions. A tutorial introduction to POP PK by Mould and Upton merits attention.^8^, ^9^

## 2 MEASUREMENT OF AND EXPLANATIONS FOR VARIABILITY: THE EXPERIMENTAL TRIAL VS. POPULATION PK

Table 1 summarizes the key differences between experimental trials and POP PK in investigating sources of PK and PK/PD variability.

**TABLE 1:** Differences between experimental trials and a population pharmacokinetics studies.

	Experimental	Population PK/PD
Conceptual frame	Specific research question; to test an hypothesis (H0)	To learn and explain
Location of study	Laboratory	Field; clinics
Subjects	Few; experimental	Many of the target population
Design	Yes (e.g., crossover)	No (observational)
Sampling	Intensive and balanced according to protocol	Sparse and unbalanced
Controlled variable	Yes (factors)	No (but covariates documented)
Status of variability (heterogeneity)	Noise except the factor under study	Potential biological information
Data analysis	Two stages	One stage (NLMEM) and data driven
Results	Mean, SD, H(0) accepted/rejected	Typical values; between‐subjects variability; subgroup identification with covariates …
Uses	For example, to compare two formulations (bioequivalence).	Dose prediction/optimization/individualization; the what if scenario; meta‐analysis, TDM …
Inference space	Narrow (possible bias)	Large

*Note*: H(0): null hypothesis.

Abbreviations: NLMEM, nonlinear mixed effect model; TDM, therapeutic drug monitoring.

The primary difference between experimental trials and pop PK/PD is conceptual: The former is designed to document some specific question and, implicitly or explicitly, to test hypotheses to answer the question. In contrast, the objective of POP PK/PD studies is to identify,^10^ with no preconceptions, sources of variability and to explain them using covariates. Arising from the conceptual difference between the two approaches, there is a major difference in the status of variability: in the experimental trial, all sources of variability apart from the “factor” under investigation by the hypothesis must be minimized or even eliminated; they constitute *noise*, which might reduce the statistical power of the test. Conversely, in a POP PK study, all biological sources of variability are considered relevant and useful items of *information*.

In an experimental trial, selection of subjects should be as homogeneous as possible, so that the research question can be addressed with a small number of animals, while ensuring the trial's power. In contrast, in a POP PK trial, the aim is to observe a maximum number of subjects that are representative of the target population in all its heterogeneity. In a POP PK trial, no factors are controlled, but potential covariates (age, sex, etc.) are documented. Finally, the inferential space, that is, the population to which the study's results apply, is narrow and focused for an experimental trial and broader and more informative for a POP PK study.

## 3 WHAT PRECISELY IS A POPULATION MODEL OR A NLMEM?

For NLMEM, mathematical models are developed allowing to estimate: (i) the so‐called *typical values* for PK and PD parameters (plasma clearance, urine‐to‐plasma ratio, EC_50_, and others); (ii) the observed variability by modeling demographic or managerial/environmental covariates; (iii) the random variability (variance within and across subjects); and (iv) a residual variability reflecting *uncertainty*. POP models contain both *fixed* and *random* effects; hence, it is a mixed‐effects model (MEM). The analysis is nonlinear (NL) MEM or NLMEM because the dependent variables (e.g., plasma drug concentration) are nonlinearly related both to the model parameters (plasma clearance, bioavailability) and independent variables (e.g., time and dosing).

**FIGURE 1:**
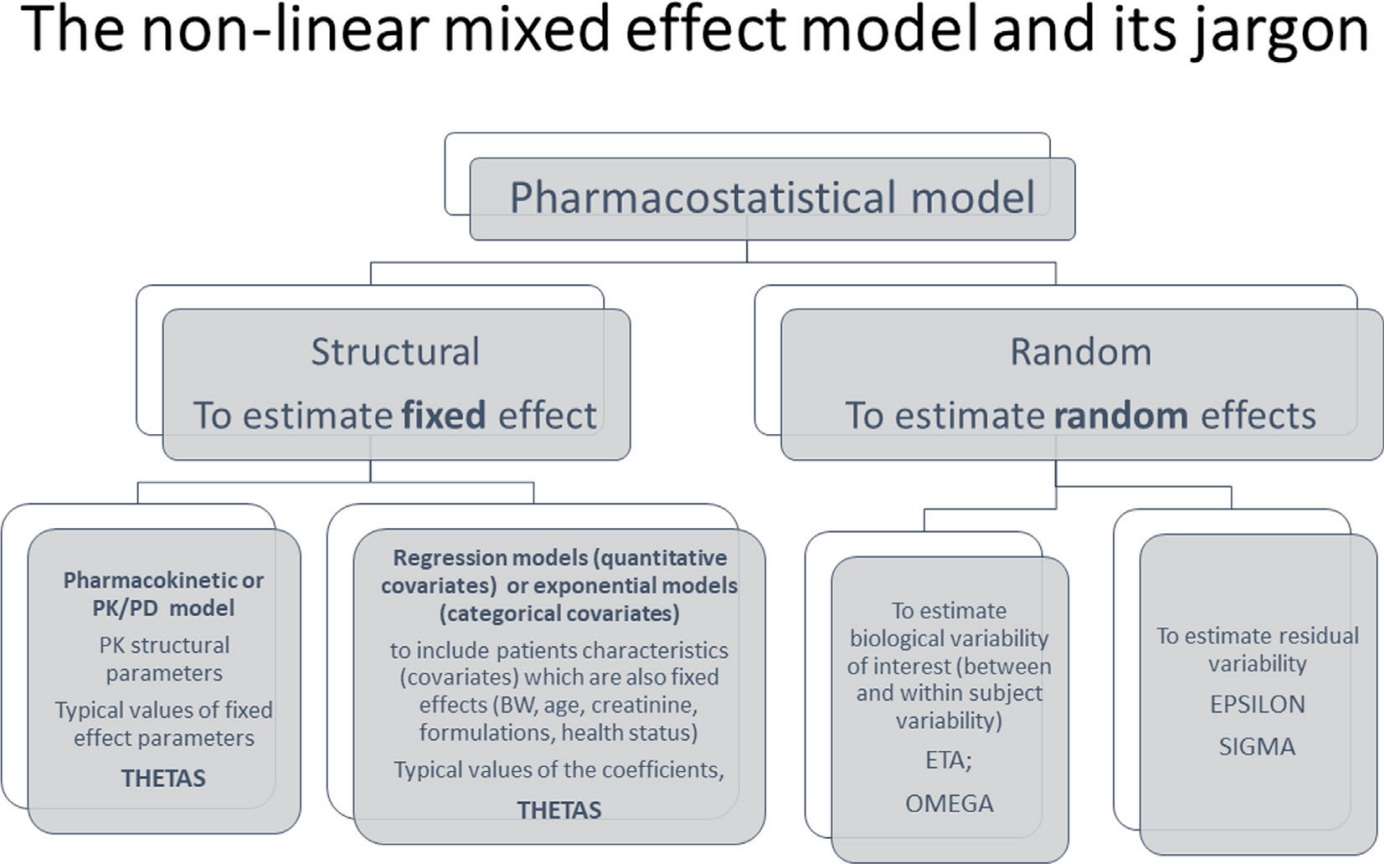
The components of a population model.

Figure 1 illustrates the components of a POP model and associated descriptors.

The pharmaco‐statistical component of POP models comprises three submodels: (i) a model for the typical response for an average subject and a set of covariates; (ii) a statistical model for heterogeneity; and (iii) a statistical model for the residual uncertainty.

The model giving the typical values for an average subject (horse) is also called the *structural model*. It is often a compartmental model, parameterized with physiologically meaningful parameters, such as clearance of elimination (to describe the elimination of the drug from the central compartment), clearance of distribution (to describe the distribution of the drug between the central and the peripheral compartments), and volumes of distribution. This enables the parameters themselves to be modeled with submodels, composed of covariates of interest. For example, the plasma clearance of a substance primarily eliminated through the kidneys, like flunixin, could be modeled with a quantitative covariate such as blood creatinine concentration, which directly reflects kidney function. The covariate model embedded in the structural compartmental model enables prediction of variations of plasma clearance, based on individual plasma creatinine concentration. This base model, together with the covariate model, captures deviations in plasma or urinary concentrations for a dosing history at a specific time. Estimated parameters (plasma clearance, regression coefficient of the covariate model for creatinine, etc.) are *fixed* effects and are noted *Thetas* in POP jargon (see Figure 1).

The second element of a NLMEM pertains to random effects, encompassing two distinct models: one for *heterogeneity* and a second for *uncertainty*. The former quantifies the extent of variability among individuals; it is noted as the between‐subject variability (BSV) or the interindividual variability (IIV). The BSV is expressed with a coefficient of variation (CV%) computed from omega, the variance of random effects. Random effects are noted ETA in pop jargon (see Figure 1). The BSV provides significant biological information. Its estimation aids in making judgments about the drug, such as classifying it as highly variable or not, determining the need to search covariates to adjust dosages, and allowing simulations and calculations of some probability of target attainment (PTA), such as population withdrawal time (WT) computations^11^ (refer to Sections 4.2 and 4.3).

As well as heterogeneity, the residual variance model aggregates all forms of uncertainty, notably analytical variability, possible dosing and sampling errors, model misspecification and, possibly, the intraindividual variability.

Intraindividual variability, the within‐subject variability (WSV), also described as interoccasion variability (IOV), refers to variations from one occasion to another within the same horse. If not documented, this source of variability is lumped in the residual variance. However, IOV warrants special attention, because it assesses the reproducibility of the parameter over time within the same horse. Evaluating IOV requires observing the same horse on different occasions. Measuring IOV is required to assess the possibility of making some Bayesian prediction to estimate an individual WT (see Section 4.3), This is because a large IOV (CV > 30%), that is, a poor repeatability of parameters from one occasion to another, renders illusory the aim of making robust individual predictions.

## 4 WHY USE POPULATION PK IN THE RACING INDUSTRY AND EQUESTRIAN DISCIPLINES?

There are many reasons for using POP PK. One is to establish the best empirical dosages for the majority of a horse population. Another is to tailor dosage for a subgroup of horses (e.g., a given breed) or for an individual horse within the scope of precision medicine. POP PK can also be used for replacing certain invasive preclinical experimental models with direct observation of the same physio‐pathological conditions under field conditions (Section 4.1). It can be further used for the determination of individualized WT, in order to ensure, with a certain statistical risk, negative results in medication controls for the individual horse (Section 4.3).

The use of NLMEM is not limited to prospective clinical trials; it can also be a crucial tool in retrospectively conducted meta‐analyses (MA) of existing but somewhat disparate data sets^12^ (Section 5). The objective here is to generate new knowledge that no single trial could have provided in isolation. It can be applied to objectively and impartially summarize the data available to groups of experts, for example, facilitating the achievement of consensus, when establishing international screening limits (SL), as conducted by organizations such as the International Federation of Horseracing Authorities (IFHA) (Sections 5.1 and 5.2).

### 4.1 POP PK applications in animal welfare, ethics and reputation of organizations

POP PK/PD modeling could contribute to presenting a positive image of the racing industry by promoting drug investigation methods that align with the 3Rs principle. The 3Rs denote Replacement, Reduction, and Refinement; they provide a framework for conducting more humane animal research. For equine medications, animal experimentation is an important component of the preclinical phase of drug development. Preclinical experimental models commonly involve inducing bacterial and viral infections or inflammation and pain induced by mechanical, thermal, or chemical irritants. They are not only commonly invasive; they are also not always firmly representative of clinical realities. Moreover, preclinical trials may require diagnostic methods that involve the euthanizing of the horse, as applies in the preclinical evaluation of antiparasitic drugs, in order to take parasite counts (adults, larvae) for the dose determination.^13^

The transfer of part of these studies currently carried out with invasive laboratory models to the observation of spontaneous clinical cases would meet the expectations of the first two R's of the 3R rule. POP PK‐PD is the tool to enable such a transition. It would bring veterinary medicine closer to the practices in human medicine, where human preclinical experimentation remains exceptional.

### 4.2 POP PK for the rational design of dosing regimens

The determination of optimal dosage of drugs involves several sequential steps. A first step is to estimate an overall dosage, broadly appropriate for the majority of horses. Second, this dose is adjusted for subgroups of horses to account, for example, for covariates such as breed, age, and health status. Third, beyond the subgroups is establishing an optimal dosage for the individual horse, within the framework of precision medicine.^14^

Dose is a hybrid PK/PD variable^15^, ^16^ calculated from its determinants (Equation 1):

(1) $$\text{Dose}=\frac{\text{Plasma clearance}\times \text{Effective plasma concentration}}{\text{Bioavailability}}$$

Using Equation (1), a dose can be calculated from the average values (point estimates) of its three determinants. This yields an “average” dose. If Equation (1) is solved with the distributions of these three determinants, a distribution of doses is obtained from which a quantile (for example Q90) can be determined and proposed as a dose to cover a majority of horses (90%) in the population targeted. This dose will be higher than the average dose because it takes into account the differing sources of variability of the three components of Equation (1).

It is only by using Monte Carlo mathematical simulation to compartmentalize the dosage and account for various covariates, such as breed, that predicted dose might differ between Thoroughbreds and Standardbreds, with age (foal vs. adult) or health status. A further refinement could be to adjust dosages to allow for horse‐specific covariate values (breed, age, weight, etc.), while analyzing two or three blood samples at the commencement of treatment from a specific horse. This approach, known as therapeutic drug monitoring (TDM), is still little used in veterinary medicine precisely because the population models required for its use have not yet been developed. The approach, which is particularly applicable to drugs with a narrow therapeutic window, constitutes *precision medicine*. For a racehorse, individual animal calibration would ensure optimal management of administered doses relative to future sporting events. It would also provide equine veterinarians with access to new generations of drugs, such as monoclonal antibodies; in human medicine, these are excellent candidates for precision medicine,^17^ especially in light of their high cost, that is, for a pharmacoeconomic rationale.^14^

This method for dose determination requires development of what is described in human medicine as model‐informed precision dosing (MIPD). Furthermore, to render these MIPDs applicable for the horse requires access to a user‐friendly and free‐to‐use package, which allows the prescriber to individualize the dose horse‐by‐horse. In human medicine, Posologyr is an example of an R package freely available and compliant with the needs of clinical providers (https://github.com/levenc/posologyr/).^18^

### 4.3 POP PK and individualization of withdrawal time

It is a requirement that a minimum delay is observed, after the administration of a medication, before a sporting event or race, to ensure that that the horse is no longer under the residual influence of the medication. Depending on individual governing bodies, this delay can be institutionally managed either through a detection time (DT) or a withdrawal time (WT).

For The European Horserace Scientific Liaison Committee (EHSLC), a DT is for information and not a recommendation. More precisely, the DT is the first observed time point at which urine and/or plasma samples collected from all investigated horses (generally *n* = 6) are below the SL.^19^ It should be noted that DT is a measure with no statistical protection; it is released by racing authorities solely to assist veterinarians in subsequently recommending their own individual WT. This explains why an individual WT, recommended by a veterinarian for horses competing in Europe, should be longer than the EHSLC's DT; that is, it is to avoid the risk of a positive test. Veterinarians should transform a DT into an individual horse WT by taking into account the impact of all possible sources of animal variability^20^ and adding to DT a safety span.^21^

In contrast, the Racing Medication & Testing Consortium (RMTC), WT can be viewed as an institutional population WT. The goal here is to protect trainers against the risk of a horse being declared positive, and hence further protecting the reputation of the racing organization. It should be understood that determining a not too long WT for which 95% of the horses are below the SL with 95% confidence has a price. The price paid is to select for control, in most instances, a higher SL than that derived using PK/PD principles.^22^

**FIGURE 2:**
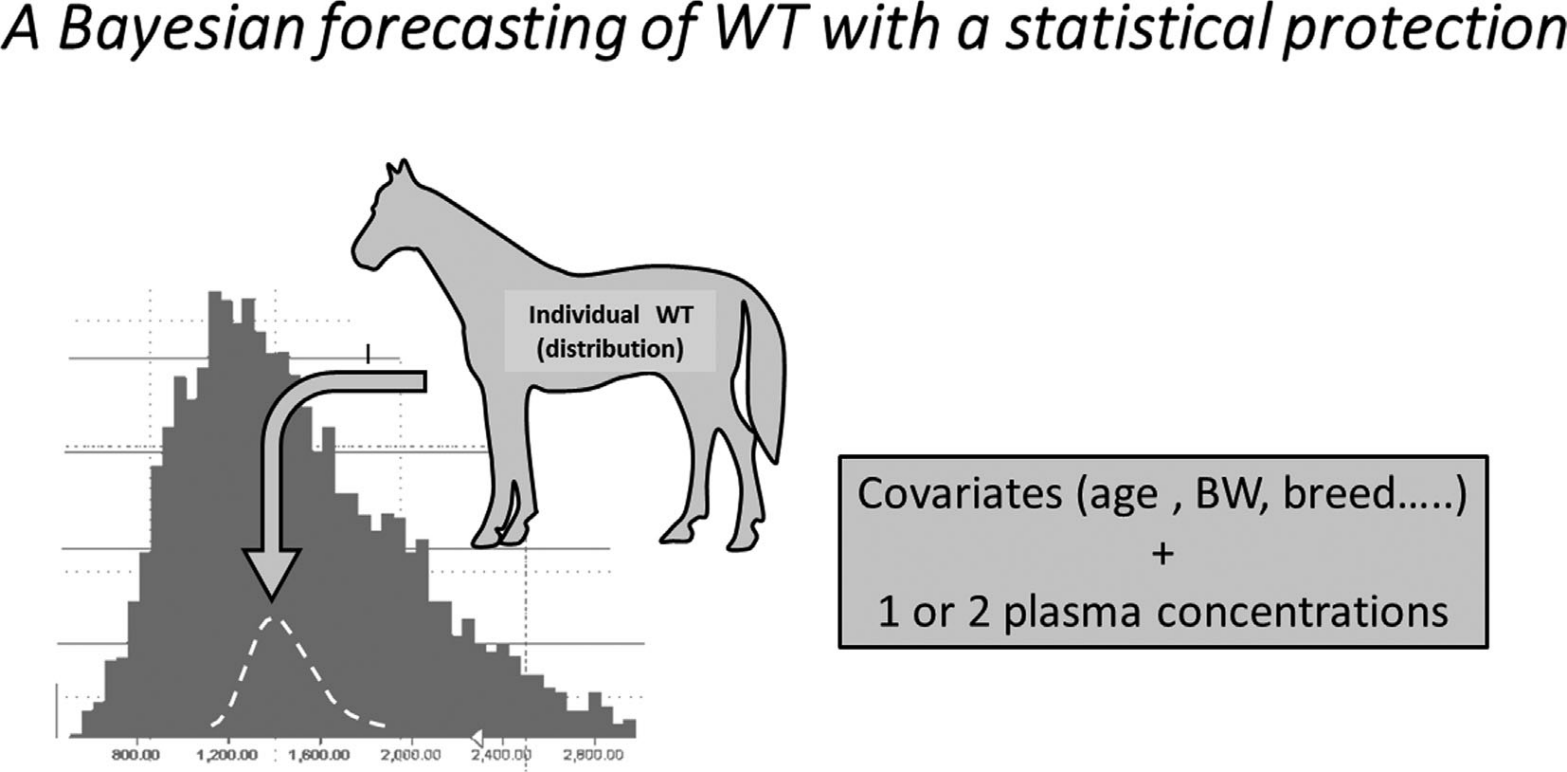
Bayesian estimate of an individual withdrawal time (IBWT). An individual WT can be estimated using an available ready‐to‐use ad hoc POP model and individual covariates for a given horse, provided that they have been effectively documented in the model (e.g., age, BW, and breed). The condition for having realistic predictions is a low inter‐occasion variability for the drug (<30%). If, in addition, the practitioner can provide one or two plasma (or urinary) concentrations, measured during a previous administration, he will be able to estimate WT of the horse in a Bayesian manner with statistical protection. With this approach, the IBWT for a few major drugs, selected from several alternatives, could be estimated for a horse to best manage its participation in future sporting events.

In order to lessen the gap between these two strategies, an individual Bayesian withdrawal time (IBWT) with its statistical protection is proposed (Figure 2).

The basic principles of the Bayesian approach in horse medication control have been proposed.^23^ With this approach, prescribers will be able to estimate the WT for a given horse under their care, that is, to calibrate prospectively the horse in question. A requirement for this is access to an ad hoc POP model, in which covariates such as breed and age are documented in the structural model and, ideally, to then collect one or two plasma samples.

For the prescriber, it would be advantageous to promote the advancement and dissemination of open‐access MIPD for the principal medications used in horse racing. In addition, some tailored open‐source and user‐friendly tool package should be developed to allow each prescriber, given a set of his/her horse‐related data from these MIPD, to estimate the most likely WT for a given horse with its statistical protection, that is, a full posterior (conditional) distribution of IBWT that retains information about the uncertainty associated with IBWT estimates.^18^

Such an IBWT would to provide European prescribers with statistical protection that they do not currently have with a DT. For US jurisdictions, it would provide a choice of the more conservative SL, in respect of animal welfare, without necessarily leading to a WT that would be deemed unacceptable by their stakeholders. Furthermore, estimating IBWT for multiple drugs with similar profiles for a particular horse could enable the prescribing veterinarian to choose between several therapeutic options, taking into account the likely associated individual WTs for future events for the horse under their care.

## 5 POP PK FOR META‐ANALYSIS (MA) OF EXISTING DATA TO GENERATE NEW DRUG KNOWLEDGE

A recent motivation in veterinary medicine for POP PK modeling has been to carry out MA of existing data.^24^ MA is the statistical procedure for combining PK data from multiple past studies conducted on a given drug. The gold standard method is the individual patient data meta‐analysis (IPDMA)^12^; that is, for the horse, this involves the reanalysis of all raw data collected in horses enrolled in several trials. For decades, the various jurisdictions have carried out numerous experimental investigations, generally on small numbers of horses and under controlled (but differing between studies) experimental conditions (route of administration, methods of administration, sampling frequency and duration, etc.), and additional experimental conditions (rest, training, etc.). Plasma and urinary concentrations were measured with analytical techniques specific to each laboratory and, indeed, which sometimes differed substantially for the same laboratory, leading to analytical performance differences over time, especially for the limits of quantification (LOQ).

Arising from progress in analytical method sensitivity has been the need to establish additional phases of drug elimination from plasma and in urine, with increased half‐life times.^25^ A negative consequence has been to render historically collected data censored and unbalanced and, de facto, unusable. A further issue arising from some specific trials, even those recently conducted, has been to render deduction of potential influences of breed, age, sex, level of training, and state of health on drug disposition not possible. An MA, in contrast, has the power to generate information that no single jurisdiction could have generated. This is explained by the size effect of the samples studied. What is difficult or even impossible to estimate on a small number of, often relatively homogeneous, horses (e.g., a breed effect) may become estimable when all data from all horses of several jurisdictions are pooled and analyzed simultaneously. A key feature of NLMEM is that all data collected, old or recent, sparse or rich, censored or not, experimental or observational, plasma, urine, or other matrix, can be analyzed simultaneously. Thus, NLMEM is the appropriate statistical tool to analyze unbalanced and censored data, while ascribing relative weights to each data set according to its quality.^7^, ^24^ The construction of this type of population model will open many opportunities for the racing industry. These will extend far beyond good therapeutic practices; as global models, they will enable the implementation of Bayesian approaches to estimation of individual WT (as explained in Section 4.3), and they will facilitate consensus in determining international SL (Section 5.1). MA has been used in studies of human doping control to assess the adequacy of the salbutamol threshold, thereby drawing the distinction between therapeutic use and violation of anti‐doping rules,^26^ and in horses to establish clinical breakpoints for interpretation of antimicrobial susceptibility testing of benzylpenicillin.^27^

### 5.1 Population modeling for MA and international screening limits harmonization

The control of medication for racing and other equine sports requires setting SLs. International organizations, such as IFHA, have the responsibility for proposing international SLs. Ideally, these should be determined as part of a sequential three‐step risk analysis (RA), namely, risk assessment, risk management, and risk communication.^28^

The first step is a scientific task; it involves inspection of plasma and urinary concentrations or corresponding PK parameters collected by the several jurisdictions. This task is rendered difficult especially if the available data are heterogeneous in their informative value (abundance and quality). Indeed, the first condition required for a group of experts, convened to discuss and decide on an international SL, is that they can collectively examine and share with identical approaches all raw data at their disposal in a format which facilitates collective discussion. An important first step will be for all available raw data having undergone an MA. This will distill all data into a form readily comprehensible and therefore readily shared by the group. It will ensure convergence of review and therefore of decision taking. The NLMEM will estimate central tendencies of interest with the computation of some “typical values” and optimally summarize all available data. An example is estimation of a “typical clearance” value in horses from which to define a “typical Effective Plasma concentration or EPC” for the substance subject to expert appraisal. As well as defining typical values of a PK parameter, the same NLMEM model will provide the best estimate of overall variances of each parameter in the horse population, ascribing to them covariates such as countries, breed, and age, provided that they have been documented in the data base. In addition, modeling will enable simulation of different scenarios to estimate future WTs, making into account covariate effects that may lead to different regional WT, depending of jurisdictions (e.g., due to a breed effect).

The second step is risk management. This involves deciding the SLs by expert consensus. Ideally, the discussion by the panel of experts on the different jurisdictional raw data sets should be limited. The focus should be on the MA findings, which lead to proposing an international SL. For institutions or organization adhering to PK/PD principles,^22^ this step operationally translates into the selection of a safety (or uncertainty) factor (SF). This factor transforms the computed EPC into an ineffective and irrelevant plasma concentration (IPC).^22^ By default, an SF of 500 has been historically proposed to transform an EPC into an IPC. This factor is itself the product of two factors: 50 and 10. The 50 factor transforms an average EPC into an average IPC, corresponding to residual effects of the medication that did not exceed a median effect by 2%, assuming that the concentration‐effect relationship obeyed a classical Emax PD model. The factor of 10 reflects both the inter‐individual variability of the PK (factor of 3.33) and that of pharmacodynamic origin (factor of 3.33) as classically conducted in toxicology studies to compute a no‐effect level.^22^ With POP PK data, these two a priori factors could be revised in light of the MA, which estimates the variances of interest, including clearance. In addition, Monte Carlo simulation of scenarios of variable complexity can furnish the expert panel with a range of possible outcomes, in terms of DT or protected WT and the probabilities that they will occur for any selected SL. International organizations will thereby be able to make more informed choices, based on a clearer understanding of the risks and uncertainties associated with each option.

### 5.2 Expert knowledge elicitation

The main obstacle to achieve rapidly a consensus (or a dissensus) is not of scientific origin but is linked to the experts themselves, to their culture, and ego. Therefore, a key question for international organizations is how to aggregate divergent expert judgments, in advising policy makers. An attractive option is to adopt Expert Knowledge Elicitation (EKE). EKE consists of assimilating knowledge and judgments from several experts with likely differing opinions. The European Food Safety Authority (EFSA) has issued a guidance on EKE.^29^ Frequently, EKE is deployed in situations where there is uncertainty or risk. There are compelling theoretical and practical arguments to conclude that the proper representation of an expert's knowledge about an uncertain quantity is a probability distribution.^30^, ^31^ The proposal is that each expert is asked to express their opinion either in terms of a probability or through lower and upper limit values with the most plausible (median) values. Then, mathematically aggregating these distributions from several experts provides the final probability distribution, which a neutral observer could accept as representative of the consensual deliberations of the experts.^29^

Free software solutions are available with varying methodologies. For example, the Sheffield methods, employing behavioral aggregation, offers a package (SHELF) that is free to download at https://shelf.sites.sheffield.ac.uk/.

## 6 STRENGTHS AND LIMITATIONS OF POP PK

The strength of POP PK is its greater relevance to the use of therapeutics in the equine population during management and participation in competitive sports, notably horse racing, than experimental studies conducted under variable and biased laboratory conditions. Furthermore, some covariates, and their possible correlations, cannot readily be studied in the laboratory setting. Much more readily and meaningfully studied in the field, for example, is the impact of health status^32^ and the horse's activity level on the distribution and elimination of drugs. A specific example is tiludronic acid; a POP PK study showed that the terminal half‐life was significantly longer in horses in training compared with resting horses (370 vs. 289 h, respectively).^33^ A consequence is that establishing a DT based on resting experimental horses would be misleading.

POP PK additionally enables MA of data, and this, in turn, reveals sources of variability that cannot be addressed in a laboratory experiment. POP PK is the only means of investigating breed effect, as it is very costly for a single jurisdiction to study all horse breeds. The problems of data comparability can be solved by MA when developing international SL, whereas POP PK facilitates expert intervention at each stage of the management steps of a risk analysis.

For the prescribing veterinarian, the main advantage of POP PK will be the provision of predictive tools to implement a more precise therapeutic approach, regarding the choice of drug and dosage, and especially to assist the clinician in determining an individually tailored WT.

The challenges for POP PK include organizational difficulties and regulatory acceptability of this method of clinical evaluation, which is not intended for drug registration purposes, through its financial cost, data reliability, and the complexity of data analysis. The methodology of clinical trials, including organizational aspects, is well known to pharmaceutical companies, and it will suffice to draw inspiration from it by following the VICH guidelines dedicated to Good Clinical Practices in the target species. The VICH document has been adopted by the EU, Japan, and the USA. This incorporates obtaining informed consent from the animal owner and submission to an ethical committee, among other stipulations. A clear conclusion is that these trials will be significantly simpler than those aimed at demonstrating the efficacy of a medication, as the primary requirement is collection of blood and urine samples from each horse. Data reliability is a crucial consideration, especially for dosage and timing of administration and sampling. Also necessary is knowledge of relevant covariates with good data traceability. This will involve training programs for investigators, who often will be prescribing veterinarians. Validating their practices and equipment, remunerating them, and conducting regular audits will also be essential features. None of these requirements and challenges are relevant to the conduct of MA modeling. In the final analysis, regardless of source (experimental, obtained during a specific clinical trial, retrieved from other trials for MA modeling), it will be necessary for data to be analyzed by professional modelers, that is, pharmacometricians. These modelers must be well versed in the principles and practice of PK, PD, and PK/PD, as well as possessing technical pharmacometrics skills, including but not limited to NLMEM. Not least, they should be effective communicators. The ideal profile for these modelers has been outlined.^34^ A positive start would be for the racing industry to sponsor PhD positions in pharmacometric teams and to train their future collaborators.

## 7 CONCLUSION

POP PK is a new scientific frontier, with multiple applications for the racing industry. Unlike other animal species, the horse and especially the racehorse is a high‐value individual for which individualized medicine can and should be achieved. It is the antithesis of mass medication, which is the norm in livestock. Moreover, the racing industry and equestrian sports are globalized activities with universally common issues managed by international organizations with the ability to comprehend the issues and the power to influence practices, which enhance animal welfare and ensure that sporting fairness is optimized.

## CONFLICT OF INTEREST STATEMENT

The author declares that there is no conflict of interest.
